# Seasonal prevalence and determinants of food insecurity in Iqaluit, Nunavut

**DOI:** 10.3402/ijch.v74.27284

**Published:** 2015-08-05

**Authors:** Yang Guo, Lea Berrang-Ford, James Ford, Marie-Pierre Lardeau, Victoria Edge, Kaitlin Patterson, IHACC Research Team, Sherilee L. Harper

**Affiliations:** 1 McGill School of Environment, McGill University, Montreal, Canada; 2 Department of Geography, McGill University, Montreal, Canada; 3 Indigenous Health Adaptation to Climate Change Research Team: Cesar Carcamo, Alejandro Llanos, Shuaib Lwasa, Didacus Bambaiha Namanya; 4 Department of Population Medicine, University of Guelph, Guelph, Canada

**Keywords:** food security, aboriginal, Inuit, Indigenous, social determinants of health, Iqaluit, Nunavut

## Abstract

**Background:**

Food insecurity is an ongoing problem in the Canadian Arctic. Although most studies have focused on smaller communities, little is known about food insecurity in larger centres.

**Objectives:**

This study aimed to estimate the prevalence of food insecurity during 2 different seasons in Iqaluit, the territorial capital of Nunavut, as well as identify associated risk factors.

**Designs:**

A modified United States Department of Agriculture Food Security Survey was applied to 532 randomly selected households in September 2012 and 523 in May 2013. Chi-square tests and multivariable logistic regression were used to examine potential associations between food security and 9 risk factors identified in the literature.

**Results:**

In September 2012, 28.7% of surveyed households in Iqaluit were food insecure, a rate 3 times higher than the national average, but lower than smaller Inuit communities in Nunavut. Prevalence of food insecurity in September 2012 was not significantly different in May 2013 (27.2%). When aggregating results from Inuit households from both seasons (May and September), food insecurity was associated with poor quality housing and reliance on income support (p<0.01). Unemployment and younger age of the person in charge of food preparation were also significantly associated with food insecurity. In contrast to previous research among Arctic communities, gender and consumption of country food were not positively associated with food security. These results are consistent with research describing high food insecurity across the Canadian Arctic.

**Conclusion:**

The factors associated with food insecurity in Iqaluit differed from those identified in smaller communities, suggesting that experiences with, and processes of, food insecurity may differ between small communities and larger commercial centres. These results suggest that country food consumption, traditional knowledge and sharing networks may play a less important role in larger Inuit communities.

Food security exists “when all people, at all times, have physical, social and economic access to sufficient, safe and nutritious food to meet their dietary needs and food preferences for an active and healthy life” (1, p. 1). Access to adequate food has been identified as a major challenge in the Canadian Arctic, where levels of food insecurity are consistently higher compared to southern Canada (2–7). Studies highlight that women, older residents, and those relying on income support are often more likely to be food insecure (8,9). Yet, having an active hunter in the household or consuming country food has been shown to be protective against food insecurity (7,9,10). Unemployment, low income, increasing cost of hunting, socio-cultural changes, such as reduced sharing of food and decreased transfer of traditional hunting knowledge, and climate change have also been identified as stressors to food systems in the Circumpolar North (2,8,10–14).

Most studies on food insecurity in Arctic Canada have focused on small, remote communities (e.g. studies conducted as part of the Healthy Foods North programme) or have examined the prevalence of food insecurity at a regional scale (e.g. the Inuit Health Survey) (4,7). While these studies have substantially contributed to our understanding of food insecurity, limited research has been conducted in larger centres of the North (15,16). These rapidly growing settlements are home to almost one fifth of all Inuit people in Canada, and differ in social-economic-demographic structure from smaller communities where research has been primarily conducted (17). As such, it is unknown if predictors of food insecurity identified in the literature are applicable to larger regional centres, where identified protective factors, including sharing networks, employment, education, and participation in traditional harvesting activities, may differ.

As pointed out by the Expert Panel on the State of Knowledge of Food Security in Northern Canada, there has also been limited research conducted on the seasonality of food insecurity (18). Scholarship on food security in the Arctic, including the 2007–2008 Inuit Health Survey, is mainly cross-sectional (6,9,19). These methodological choices matter because the timing of ice break-up and freeze-up, and weather conditions influence the distribution and accessibility of harvesting sites, ultimately affecting the type and quantity of food consumed (20,21). For instance, data on country food harvest in Iqaluit indicate seasonal variation in caribou, ringed seal and Arctic char harvesting rates (22). Only a few studies, however, have assessed the composition of the seasonal diet of Indigenous people in Canada. Kuhnlein et al. (23) conducted dietary assessments in 44 communities (Inuit, Dene/Métis and Yukon First Nations) during a season of high and low traditional food availability. Other studies conducted in Baffin Island, Yukon, and the Inuvialuit Settlement Region found significant seasonal variation in availability or consumption of traditional food (13,24).

In this study, we report on a seasonal analysis of household food insecurity and associated determinants in Iqaluit, Nunavut (population of 6,699) (25). As the capital and largest city of Nunavut, Iqaluit is the seat of many governmental agencies and Inuit organizations. Compared to small villages in the Canadian Arctic, Iqaluit has a strong wage economy and attracts a large number of external workers, both Inuit from other Arctic communities and non-Indigenous migrants from southern regions (26). Despite its size and function, there are little data on food insecurity in Iqaluit specifically, with previous research either focusing on particular groups (e.g. community food programmes) or on Nunavut as a whole (7,15,16). The goal of this study was therefore to estimate the prevalence of food insecurity in Iqaluit, as well as the prevalence and predictors of food insecurity for Inuit households in 2 different seasons.

## Methods

**Fig. 1:**
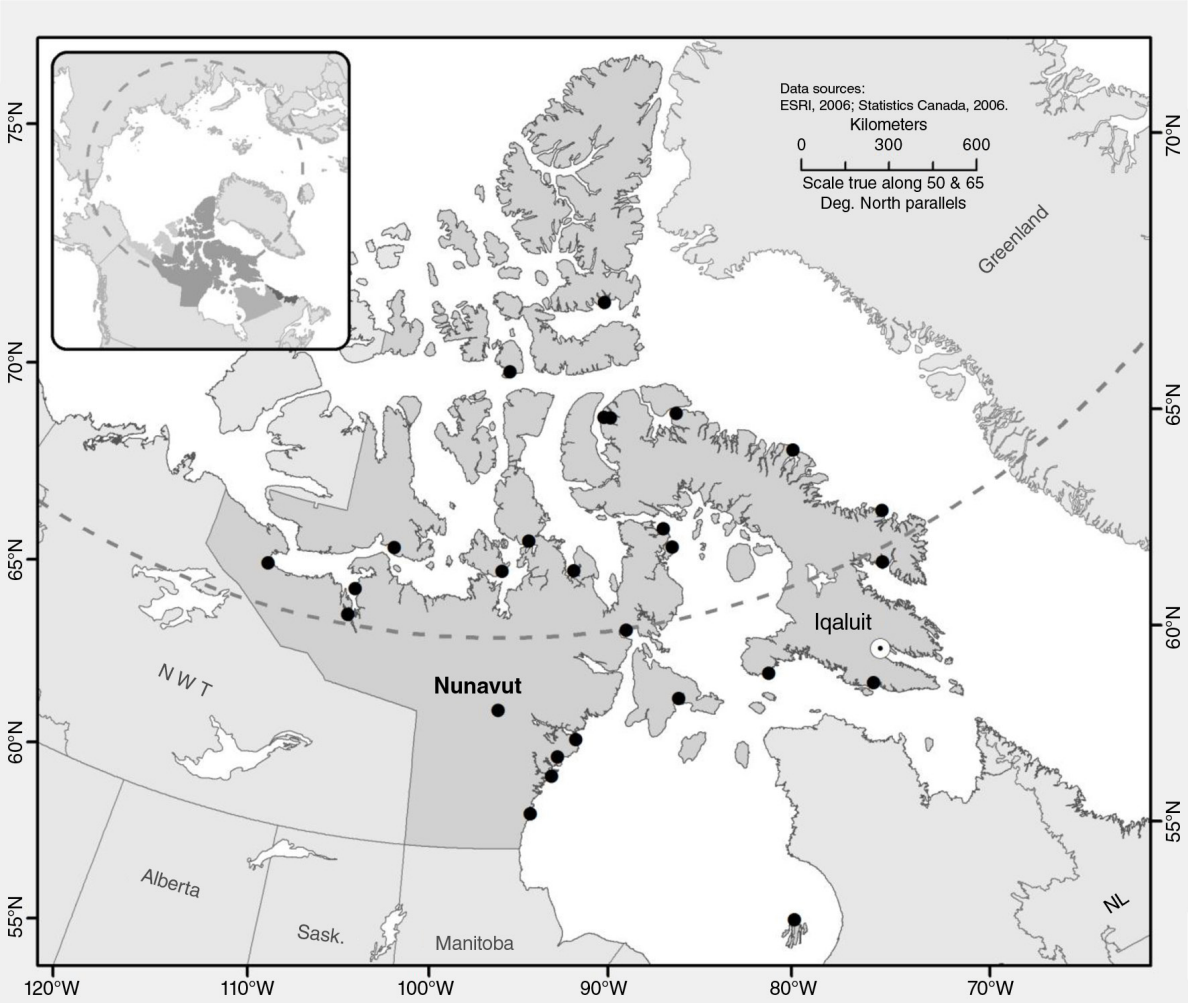
The Canadian Territory of Nunavut with Iqaluit highlighted.

Data were collected using a repeated randomized cross-sectional household survey conducted in Iqaluit from September 15th to October 5th 2012 and from May 18th to May 31st 2013 (Fig. 1). The open water boating season in Iqaluit typically runs from late June/early July to November (21). Outside of this period, the use of a snowmobile to go out on “the land” is preferred due to stable ice conditions and extensive snow cover (21). The 2 survey periods were thus chosen to reflect a period of potentially low and high accessibility to country food harvest, respectively. May is part of the “shoulder season,” a period of heightened vulnerability due to spring ice break-up (21). Transient food insecurity can occur during this period, as access to hunting grounds (both by boat and snowmobile) becomes limited (12). September/October was chosen as a period of high accessibility to harvesting areas as it occurred during the boating season, before the winter ice freeze-up (21).

During both seasons, the City of Iqaluit’s House Number Atlas was used to select households by dividing the city area into 4 neighbourhoods, based on shared characteristics and geographical location. Each neighbourhood was then further divided into map components (or “blocks”), for a total of 18. “Blocks” within each neighbourhood were proportionally and randomly selected for surveyors to subsequently visit. The process of random selection was done separately for each season and was thus independent. We surveyed all households in the block sample, both Inuit and non-Inuit. An individual from each household was randomly selected, based on the person with the most recent birthday, to answer questions about country food consumption. These questions were part of a larger survey on acute gastrointestinal illnesses, for which a random sample was required (27). Questions about food security were answered by the adult (>18 years old) in charge of food preparation, regardless of date of birth (Table I) (28). Questions about food security as well as individual-level questions were answered by the person in charge of food preparation for the household, regardless of date of birth. The ethnic origin of the household was determined by the ethnic origin of the person in charge of food preparation. Although food security status was measured at the household level, predictor variables were measured at both the household and individual levels, consistent with methods used in other Inuit focused food security studies (5,7). Ethics approval was obtained from the Research Ethics Boards of McGill University (REB: 180-1212) and the University of Guelph (REB: 11JL004). A research license from the Nunavut Research Institute was also issued (REB: 01 014 13R-M).

Overall, 532 and 523 respondents were interviewed in September and May, respectively. The crude response rate was 75% in September and 55% in May. The lower response rate in May might possibly be due to more people going out on the land in the weeks preceding the spring break-up. Surveys were predominantly conducted face-to-face by the survey team, which consisted primarily of local Inuit research assistants, and some southern-based university students, healthcare practitioners, and academics. Respondents were given the choice of completing the questionnaire in English, Inuktitut, or French. A small number of questionnaires were completed via telephone if the respondent was unavailable to answer the questionnaire in person at the time and requested a telephone survey (9.2% in September and 17.5% in May). During both seasons, surveys were performed using an iPad-based application, iSurvey (version 2.8.3). A $20 Canadian dollar (CAD) gift card for local food stores or gas stations was provided as compensation to respondents along with a ticket for a larger prize draw, as per guidelines for conducting research in northern settings.

To estimate food security status, we used a food security questionnaire with a modified recall period based on the United States Department of Agriculture (USDA) Food Security Survey Module (FSSM) (29). The FSSM is a validated and widely used tool to measure food security (30). The module contains 10 standard questions and an additional 8 questions asked if children (<18 years old) are living in the household. In contrast to previous northern food security research that used a 12-month recall period (5,19,31), but consistent with other studies (32,33), we employed a recall period of 1 month. This shorter recall period allowed for repeated sampling and assessment of seasonality. More importantly, discussion with local residents and decision makers revealed concerns over asking questions based on a 12-month recall period, which was believed to be too long. This recall period is similar to the methodology of other studies, such as work done in Toronto, as well as in the USDA Reports on Household Food Security (32,33). Several Arctic studies and the Canadian Community Health Survey use a 12-month recall period, however, and must be considered when comparing this study with previous work.

Food security status was determined using the USDA classification (Appendix A). Each household was given a score that represented the number of affirmative (positive) responses from the FSSM (29). Positive answers were coded 1 and negative answers were coded 0 and were then totalled (Appendix B). The household score was then converted to a code ranging from 0 to 3, based on a scale of severity of food insecurity. A food secure household was coded as 0 or 1, while a food insecure household was coded as 2 or 3 (Appendix A).

Pearson’s chi-squared test of independence was used to verify the association between food security status and 9 respondent characteristics previously identified in the literature as food security risk factors in the Canadian Arctic (Table I). For each test, food security was defined as a dichotomous variable reflecting food secure versus food insecure, with the food secure category including 2 levels of food security (“high” and “marginal”), and the food insecure category including 2 levels of food insecurity (“low” and “very low”).

**Table I:** Variables included in data analysis as potential predictors of food security among Inuit respondents in Iqaluit, Nunavut, in September 2012 and May 2013

	Predictor	Description	Justification	Type
Individual-level questions	Age	Age of the person in charge of food preparation	Elderly respondents might be more food secure due to better budget management skills (8)	Categorical
	Sex	Sex of the person in charge of food preparation	Women are hypothesized to experience higher food insecurity (9, 12)	Categorical/dichotomous
	Formal education level	Highest level of formal education attained by the person in charge of food preparation	Higher formal education level has been associated with reduced food insecurity (31)	Categorical/ordinal
	Employment status	Current employment status of the person in charge of food preparation	Employment has been associated with reduced food insecurity (8)	Categorical/ordinal
Household-level	Presence of child in household	Presence of a person under the age 18 currently residing in the household	Households with children experience higher food insecurity (5, 31)	Categorical/dichotomous
questions	Consumption of country food	Frequency of consumption of country food in the last month of the person who had the most recent birthday	Respondents who regularly consumed country food were less likely to be food insecure (9)	Categorical/ordinal
	Presence of mould and/or major repairs required	Whether the house had a problem with mould and/or was in need of major repairs	Respondents who live in a house requiring major repairs were more likely to experience food insecurity. Mould was also tested in the model (7)	Categorical/dichotomous
	Reliance on income support	Whether any member in the household received income support in the past month (Government of Nunavut income support programme)	Households that rely on income support experience higher food insecurity (7)	Categorical/dichotomous
–	Season	The season during which the respondent was surveyed (September–October or May–June)	Country food availability varies with season in various Indigenous communities (13)	Categorical/dichotomous

Multivariable logistic regression was used to identify significant associations between food insecurity and potential risk factors. Three multivariable models were built: (a) September 2012 model, (b) May 2013 model, and (c) a model with aggregated September and May data. For each model, a best-fit model was built using a manual iterative backward stepwise elimination procedure to identify key predictor variables. Selection used a preliminary significance of α>0.20. Spearman’s rank correlation was used to determine correlation between predictor variables to avoid collinearity problems in the models. Predictor variables that were strongly correlated with each other were removed and the more significant variable was retained. Results were considered statistically significant at α=0.05. Lowess smoothing was used to visually verify linearity between age and food security. Variance inflation factors (VIF) for each model were examined, and Akaike’s and Bayesian information criteria were used to build our best-fit model. We graphically assessed standardized and Pearson residuals, influence by delta-beta, and leverage using scatterplots. Any covariate patterns showing unusual values were noted, and models were rerun without these covariate patterns to assess any changes in the coefficients and p values in the model to ensure that the models were appropriately specified and well-fit. Data were imputed in Microsoft Excel (Version 12.0) and tests conducted using Stata/SE (Version 13.0).

## Results

### September 2012

Forty-two of the 532 September household questionnaires were removed because the respondent in charge of food preparation respondent did not answer the food security section of the questionnaire (either by choice or due to unavailability). Forty-four additional questionnaires were removed because of incomplete answers which could not be imputed based on the model from Bickel et al. (28). Overall, 446 participant responses (26% of all households of Iqaluit) from September were retained for analysis (adjusted response rate of 64%) (25). Among the 446 participants who answered the FSSM, 281 (63%) of respondents were female and 165 (37%) were male. Two hundred sixty-eight (60%) self-identified as Inuit and 178 (40%) self-identified as non-Inuit.

Among the 446 households (Inuit and non-Inuit), 318 (71.3%) were food secure and 128 (28.7%) were food insecure (Table II). The proportion of food insecure households is more than 3 times higher than the Canadian average (8.3%) (34). The level of food insecurity in Iqaluit were, however, lower compared to the Nunavut average (36–69%), including Igloolik (64%) and Kugaaruk (83%) (3,6,9,34). Prevalence of food insecurity in our study was also lower compared to the Canadian Arctic average as measured by the Inuit Health Survey (63%) (7).

**Table II:** Food security status of Iqaluit, Nunavut, respondents in September 2012 and May 2013^a^

		September 2012				May 2013		
				
Food security status	n	All households	Inuit respondents	Non-Inuit respondents	n	All households	Inuit respondents	Non-Inuit respondents
High food security	286	64.1 (59.7–68.6)	44.9 (38.9–50.9)	92.8 (89.0–96.6)	297	65.6 (61.2–70.0)	44.0 (37.9–50.1)	94.4 (91.2–97.7)
Marginal food security	32	7.2 (4.8–9.6)	10.5 (6.8–14.2)	2.2 (0–4.4)	33	7.3 (4.9–9.7)	11.2 (7.4–15.2)	2.0 (0–4.0)
Low food security	57	12.8 (9.7–15.9)	20.2 (15.4–25.1)	3.3 (0–6.0)	51	11.3 (8.3–14.2)	19.1 (14.2–23.9)	1.6 (0–3.2)
Very low food security	71	15.9 (12.5–19.3)	24.3 (19.2–29.5)	1.7 (0–3.6)	72	15.9 (12.5–19.3)	25.7 (20.3–31.1)	2.0 (0–4.0)

a Values are percentages (95% CI).

Percentages may not add to 100% due to rounding off.

### May 2013

For the May 2013 data, 33 of the 523 questionnaires were removed, as the food security section was not completed. Thirty-seven additional questionnaires were excluded as they had incomplete responses that could not be imputed, leaving 453 participants responses (26% of all) for analysis (adjusted response rate of 49%) (25).

Among the 453 participants who answered the food security section of the questionnaire, 275 (61%) were female and 178 (39%) were male. Two hundred fifty-four (56%) self-identified as being Inuit and 199 (44%) as non-Inuit. Prevalence of food insecurity was similar to the September results and lower compared to other Arctic communities: 330 (72.8%) of households (Inuit and non-Inuit) were considered food secure and 123 households were categorized as food insecure (27.2%) (Table II) (34).

### Characteristics of food insecurity in Iqaluit

**Fig. 2:**
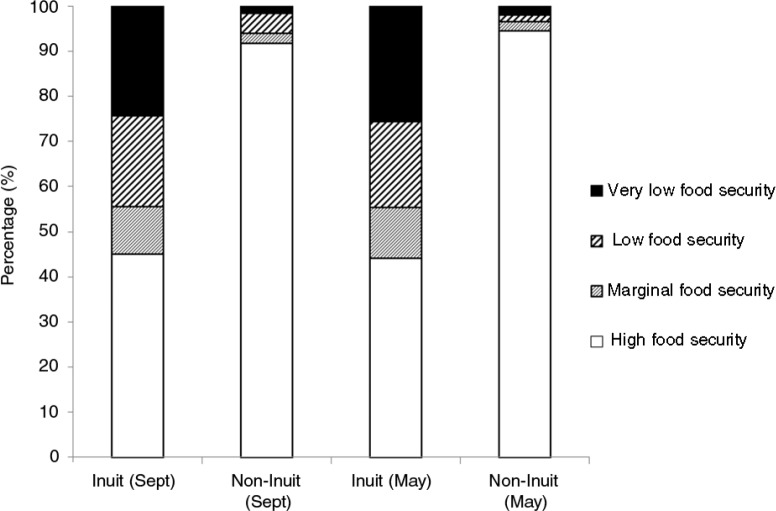
Food security status of respondents based on ethnic origin, in September 2012 and May 2013.

Food insecurity in general and more severe food insecurity in particular were more prevalent among Inuit households completing the survey than non-Inuit households (p<0.05, Tables II). Approximately 45% of Inuit households surveyed in both September and May were considered food insecure compared to only 5% of non-Inuit households in September and 4% in May (Fig. 2). Due to the low number of non-Inuit respondents categorized as food insecure (n=8 in September, n=7 in May), and also our expectation that processes and predictors of food insecurity may be patterned by ethnicity, we restricted univariate and multivariable analysis to Inuit respondents only (Table III and IV).

**Table III:** Chi-squared results of predictors of food insecurity, Inuit respondents only, in Iqaluit, Nunavut, September 2012^a^

	Total	Food secure	Food insecure	Probability (chi-squared)
Age	264 (100)	145 (55)	119 (45)	0.986
0–21 years old	28 (100)	15 (54)	13 (46)	
21–40 years old	103 (100)	57 (55)	46 (45)	
41+ years old	133 (100)	73 (55)	60 (45)	
Sex	268 (100)	148 (55)	120 (45)	0.764
Male	89 (100)	48 (54)	41 (46)	
Female	179 (100)	100 (56)	79 (44)	
Education	267 (100)	147 (55)	120 (45)	<0.01
High school not completed	168 (100)	79 (47)	89 (53)	
High school completed	50 (100)	28 (56)	22 (44)	
College or above	49 (100)	40 (82)	9 (18)	
Employment	267 (100)	147 (55)	120 (45)	<0.01
Full-time	93 (100)	71 (76)	22 (24)	
Part-time	11 (100)	7 (64)	4 (36)	
Unemployed	163 (100)	69 (42)	94 (58)	
Presence of a child (<18 years) in household	268 (100)	148 (55)	120 (45)	0.898
Yes	144 (100)	79 (55)	65 (45)	
No	124 (100)	69 (56)	55 (44)	
Country food consumption (meat from land and/or freshly caught fish in half or more of all meals)	265 (100)	145 (55)	120 (45)	0.872
Yes	109 (100)	59 (54)	50 (46)	
No	156 (100)	86 (55)	70 (45)	
Poor housing conditions (mould and/or major repairs)	258 (100)	142 (55)	116 (45)	<0.01
Yes	83 (100)	28 (34)	55 (66)	
No	175 (100)	114 (65)	61 (35)	
Reliance on income support	266 (100)	147 (55)	119 (45)	<0.01
Yes	95 (100)	25 (26)	70 (74)	
No	171 (100)	122 (71)	49 (29)	
Season	522 (100)	286 (55)	236 (45)	0.838
September–October	268 (100)	148 (55)	120 (45)	
May	254 (100)	138 (54)	116 (46)	

a Values are number of respondents (percent).

**Table IV:** Chi-squared results of predictors of food insecurity, Inuit respondents only, in Iqaluit, Nunavut, May 2013^a^

	Total (%)	Food secure (%)	Food insecure (%)	Probability (Chi-squared)
Age	252 (100)	136 (54)	116 (46)	0.811
0–20 years old	34 (100)	17 (50)	17 (50)	
21–40 years old	98 (100)	55 (56)	43 (44)	
41+ years old	120 (100)	64 (53)	56 (47)	
Sex	254 (100)	138 (54)	116 (46)	0.925
Male	89 (100)	48 (54)	41 (46)	
Female	165 (100)	90 (55)	75 (45)	
Education	254 (100)	138 (54)	116 (46)	0.018
High school not completed	143 (100)	67 (47)	76 (53)	
High school completed	64 (100)	43 (67)	21 (33)	
College or above	47 (100)	28 (60)	19 (40)	
Employment	254 (100)	138 (54)	116 (46)	<0.01
Full-time	87 (100)	66 (76)	21 (24)	
Part-time	16 (100)	7 (44)	9 (56)	
Unemployed	151 (100)	65 (43)	86 (57)	
Presence of a child (<18 years) in household	254 (100)	138 (54)	116 (46)	0.807
Yes	149 (100)	80 (54)	69 (46)	
No	105 (100)	58 (55)	47 (45)	
Country food consumption (meat from land and/or freshly caught fish in half or more of all meals)	252 (100)	137 (54)	115 (46)	0.422
Yes	81 (100)	47 (58)	34 (42)	
No	171 (100)	90 (53)	81 (47)	
Poor housing conditions (mould and/or major repairs)	250 (100)	137 (55)	113 (45)	<0.01
Yes	82 (100)	31 (38)	51 (62)	
No	168 (100)	106 (63)	62 (37)	
Reliance on income support	254 (100)	138 (54)	116 (46)	<0.01
Yes	92 (100)	26 (28)	66 (72)	
No	162 (100)	112 (69)	50 (31)	

a Values are number of respondents (percent).

### Risk factors for food insecurity among Inuit households

The prevalence of food insecurity and the risk factors associated with food insecurity did not vary between September and May. As such, the model that aggregated the September and May data is presented. In the best-fit model, food insecurity was associated with households living in poor quality housing and relying on income support (Table V). Increased age of respondents in charge of food preparation (aged 41 and older) was associated with lower odds of food insecurity than respondents in younger age groups (0–20 and 21–40 years old). Employment status of the person in charge of food preparation was also associated with lower odds of food insecurity. Season was not significantly associated with food insecurity and did not change or confound the other variable coefficients or p values in the model. However, we forced the season variable into the model to reflect the structure of our dataset. Post-estimation diagnostics indicated that the model was a good fit for the data.

**Table V:** Logistic regression models results in both seasons (September 2012 and May 2013), Inuit respondents only^a^ in Iqaluit, Nunavut

Multivariable Logistic Regression ModelOutcome: Food secure status	Model of best fit
Number of observations	501
Pseudo R^2^	0.18
Age	
Respondents age 0–20	0.35* (0.15–0.83)
Respondents age 21–40	0.43* (0.24–0.76)
Respondents age 41 and older	ref.
Person responsible for food preparation is employed	2.19* (1.40–3.43)
Presence of mould in house/major repairs required	0.42* (0.27–0.64)
Reliance on income support	0.25* (0.16–0.39)
Season	
September 2012	ref.
May 2013	0.95 (0.64–1.42)

* p < 0.01.

a Values are odds ratio (95% CI).

## Discussion

As one of the first published studies to examine the prevalence of food insecurity specifically in Iqaluit, Nunavut, this paper contributes to a nascent scholarship focusing on food insecurity in the larger, rapidly growing settlements of the Canadian Arctic. While the magnitude of food insecurity documented here is lower than results presented in previous work focusing on smaller communities and from regional studies, the prevalence of food insecurity is still higher than in southern Canada (Table VI). This result indicates that food insecurity remains a problem even in large Canadian northern communities. Food insecurity was strongly influenced by ethnic origin, with the percentage of food insecure Inuit households being 9 times higher than non-Inuit households in September 2012 and 11 times higher in May 2013. Indeed, when Inuit households alone were included in the analysis, the prevalence of food insecurity was closer to that documented elsewhere in Nunavut. This prevalence of food insecurity was particularly high given the strong economic growth in Iqaluit associated with resource development, government and associated services, and was consistent with research on Iqaluit food programmes, which has identified a chronically food insecure subset of Iqaluit’s population who has been unable to benefit from economic development (16). While we do not examine the underlying causes of such trends here, other work has identified acculturative stresses associated with community relocation, environmental dispossession, and often, forced cultural assimilation (e.g. through residential schools), as important underlying stresses facing contemporary Inuit settlements, and which frames low rates of educational attainment, higher unemployment and food security challenges (11). Indeed, as Wakegjijig et al. (14) note, to achieve any kind of success in the North, food policy must take into account these broader determinants.

**Table VI:** Published prevalence of food insecurity in communities/regions of Canada

Area of study	Prevalence of food insecurity (%)	Recall period	Survey month	Target population	Survey used	Year of survey	Author(s), year of publication
Kugaaruk, Nunavut	83 (adult) 82 (child)	12 months	October–November 2011	Inuit	Modified US FSSM	2001	Lawn & Harvey, 2003 (3)
Nunavut	69.6 (household) 56.1 (child)	12 months	Summer and fall 2007, 2008	Inuit	Modified US FSSM	2007–2008	Egeland et al., 2010 (5)
Nunavut	68.8	12 months	Summer and fall 2007, 2008	Inuit	Modified US FSSM	2007–2008	Rosol et al., 2011 (6)
Igloolik, Nunavut	64	12 months	Summer 2007	Iglulingmiut	Modified US FSSM	2007	Ford & Berrang–Ford 2009 (9)
36 communities of the Inuvialuit Settlement Region, Nunavut and Nunatsiavut	62.6	12 months	Summer and fall 2007 and 2008	Inuit	Modified US FSSM	2007–2008	Huet et al., 2012 (7)
Inuvialuit Settlement Region	43.3	12 months	Summer and fall 2007, 2008	Inuit	Modified US FSSM	2007–2008	Rosol et al., 2011 (6)
Kangiqsujuaq, Nunavik	40 (adult) 40 (child)	12 months	May–June 2002	Inuit	Modified US FSSM	2002	Lawn & Harvey, 2004 (19)
Iqaluit, Nunavut	28.7 (September) 27.2 (May)^a^	1 month	September–October 2012, May 2013	Inuit and Non-Inuit	Modified US FSSM	2012–2013	This paper
Nunavut	36.2	12 months	January 2011 to December 2012 (ongoing)	Population of Nunavut	Modified US FSSM	2011–2012	Statistics Canada 2013 (34)
Canadian average	8.3	12 months	January 2011 to December 2012 (ongoing)	Canadian Population	Modified US FSSM	2011–2012	Statistics Canada 2013 (34)

a Among Inuit only, prevalence is 44.7 and 45.7% in September and May, respectively.

Poor quality housing, unemployment and reliance on income support were associated with food insecurity for Inuit households. These factors usually indicate a lower socio-economic status, reducing households’ ability to afford fresh nutritious food (7). The Inuit Health Survey also found a higher prevalence of food insecurity in individuals living in a house requiring major repairs (7). Additionally, work done in the Canadian Arctic and across Canada indicated that households relying on income support exhibited higher prevalence of food insecurity (7,31). Unemployment is often associated with household food insecurity, although this has only been shown in communities outside of the Canadian Arctic (35). Older age was also significantly associated with food security, which might reflect better budget management skills (8).

We did not find gender or country food consumption to be associated with improved food security among Inuit respondents, contrary to previous work done elsewhere in the North (9). The lack of association between gender and food security status could potentially be explained by the format of the surveys. The questionnaire inquired about food security at the household level rather than at the individual level, which may or may not represent the food security status of the person in charge of food preparation.

The finding that consumption of country food was not associated with a food secure status supports the idea of a “nutritional transition” taking place in the Canadian Arctic from traditional foods to store-bought foods, especially in younger generations and in the larger Inuit settlements (36). Moreover, given a high degree of transience in residence, in-migration from other communities, and the size of Iqaluit, it has been argued that food sharing is practiced less often compared to smaller settlements, such that traditional foods are not necessarily available when people do not have the funds to access store-bought foods (15,16), an important and widely documented coping mechanism in smaller communities. A third explanation is that despite the continued importance of hunting and fishing in Iqaluit, engagement in these activities is proportionally lower than in smaller communities due to the strong wage economy (37). Nonetheless, wage workers in Iqaluit are still able to access country food through purchase rather than direct harvesting, which reflects yet another difference with smaller communities (38). Finally, food security prevalence and associated risk factors in Iqaluit did not differ by season. Again, unlike smaller communities, Iqaluit’s economy is primarily wage-based and less dependent on hunting and other harvesting activities, which are heavily influenced by climatic conditions (37). The results provide timely insights for food policy in Nunavut, which has been identified as a priority by different levels of government, communities, and activists (14,39) and emphasizes the unique needs of, and differences faced by, larger settlements.

Food insecurity remains a critical issue in Iqaluit. Future policies and programmes need to consider identified risk factors, taking into account the distinct needs and challenges faced by urban food insecure households. Specifically, interventions to improve financial accessibility to food, such as the Nunavut Food Security Coalition’s Nunavut Food Security Strategy and Action Plan 2014–2016, have specific importance. Indeed, two of the Coalition’s goals are to “help align income assistance food allowances with the cost of living in Nunavut” and “further develop the priority of instilling self-reliance among Public Housing tenants” (39). The Government of Nunavut’s new mandate, Sivumut Abluqta, also aims to improve public housing and income support to Nunavummiut (40). Such efforts have particular relevance for food security as they take place within the broader context of reducing poverty and rely on collaboration between different governmental agencies, not just public health. As the Coalition states “[food insecurity] is larger than the mandate of any one organization. A collaborative approach is essential” (39).

There are several limitations to this study. First, the food security questions were household-specific, while some predictor variables (such as ethnic origin) were measured primarily – and necessarily in most cases – at the individual level. As such, the socio-economic characteristics of the person in charge of food preparation might underestimate that of the household, as that person is more likely to stay at home and not be formally employed, while other household members might be. This bias is a challenge faced not only by this study, but also by observational studies in general using similar household-level standardized surveys. Moreover, predictor variables that evaluated food quality or attributes of traditional food systems such as food sharing were not included, which might underestimate food insecurity.

## Conclusion

The prevalence of food insecurity in Iqaluit was more than 3 times higher than the Canadian average and was strongly patterned by ethnic origin. These results highlight the persistence of socio-ethnic gradients in food insecurity in the Northern Canada and suggest that the factors that affect the vulnerability of households to food insecurity may be different between large and small Inuit communities in Canada.

## Authors’ contributions

Yang Guo co-analyzed data, interpreted results, prepared the primary manuscript draft, and led the editing. Dr. Lea Berrang-Ford conceptualized the project, co-designed the questionnaires, co-supervised analyses, contributed to interpretation of results, and contributed to manuscript writing. Dr. James Ford conceptualized the project, contributed to community-based research collaborations, contributed to interpretation of results, and contributed to manuscript writing. Marie-Pierre Lardeau contributed to analyses and interpretation of results, as well as manuscript editing. Dr. Victoria Edge contributed to community-based research collaborations, contributed to planning and facilitation of data collection, and contributed to manuscript editing. Kaitlin Patterson contributed to data cleaning and analyses. Dr. Sherilee Harper co-designed the questionnaires, contributed to community-based research collaborations, led data collection, co-supervised data analyses and contributed to manuscript editing.

## Conflict of interest and funding

The study design and conduct were independent of the funding source. This work was supported by the Public Health Agency of Canada; the Nasivvik Centre for Inuit Health and Changing Environments; the International Research Initiative on Adaptation to Climate Change (IRIACC) funded by the International Development Research Centre (IDRC), Canadian Institutes for Health Research (CIHR), Social Sciences and Humanities Research Council (SSHRC), and National Science and Engineering Research Council (NSERC); and a Vanier Canada Graduate Scholarship to S. Harper (CIHR).

## Appendix A: USDA Food Security Survey Module: Food security scale values and status levels

**Table:**

Food security status level			Score^a^	
	
General category	FSSM Category	Code	Households with children (out of 18)	Households without children (out of 10)
Food secure	High food security	0	0	0
Marginal food security	1	1–2	1–2
Food insecure	Low food security	2	3–7	3–5
Very low food security	3	8–18	6–10

a Values are number of affirmative responses.

## Appendix B: Database and food security coding of Food Security Survey Module answers

**Table:**

	FSSM answer	Database coding	Food security coding
Positive	Yes	1	1
	Often	1	
	Sometimes	2	
	Almost every day of the month	1	
	About half the days during the month	2	
Negative	Never	3	0
	No	0	
	A few days during the month	3	

## References

[1] FAO (2009). Declaration of the World Summit on Food Security. Proceedings of World Summit on Food Security.

[2] Myers H, Powell S, Duhaime G (2004). Setting the table for food security: policy impacts in Nunavut. Can J Native Stud.

[3] Lawn J, Harvey D (2003). Nutrition and food security in Kugaaruk, Nunavut.

[4] Sharma S, Gittelsohn J, Rosol R, Beck L (2010). Addressing the public health burden caused by the nutrition transition through the Healthy Foods North nutrition and lifestyle intervention programme. J Hum Nutr Diet.

[5] Egeland GM, Pacey A, Cao Z, Sobol I (2010). Food insecurity among Inuit preschoolers: Nunavut Inuit Child Health Survey, 2007–2008. Can Med Assoc J.

[6] Rosol R, Huet C, Wood M, Lennie C, Osborne G, Egeland GM (2011). Prevalence of affirmative responses to questions of food insecurity: International Polar Year Inuit Health Survey, 2007–2008. Int J Circumpolar Health.

[7] Huet C, Rosol R, Egeland GM (2012). The prevalence of food insecurity is high and the diet quality poor in Inuit communities. J Nutr.

[8] Chan HM, Fediuk K, Hamilton S, Rostas L, Caughey A, Kuhnlein H (2006). Food security in Nunavut, Canada: barriers and recommendations. Int J Circumpolar Health.

[9] Ford JD, Berrang-Ford L (2009). Food security in Igloolik, Nunavut: an exploratory study. Polar Rec.

[10] Ford JD, Smit B, Wandel J (2006). Vulnerability to climate change in the Arctic: a case study from Arctic Bay, Canada. Global Environ Chang.

[11] Richmond CA, Ross NA (2009). The determinants of First Nation and Inuit Health: a critical population health approach. Health Place.

[12] Beaumier M, Ford JD (2010). Food insecurity among Inuit women exacerbated by socio-economic stresses and climate change. Can J Public Health.

[13] Wesche SD, Chan HM (2010). Adapting to the impacts of climate change on food security among Inuit in the Western Canadian Arctic. Ecohealth.

[14] Wakegijig J, Osborne G, Statham S, Issaluk MD (2013). Collaborating toward improving food security in Nunavut. Int J Circumpolar Health.

[15] Lardeau M-P, Healey G, Ford J (2011). The use of photovoice to document and characterize the food security of users of community food programs in Iqaluit, Nunavut. Rural Rem Health.

[16] Ford J, Lardeau M-P, Vanderbilt W (2012). The characteristics and experience of community food program users in Arctic Canada: a case study from Iqaluit, Nunavut. BMC Public Health.

[17] Statistics Canada (2006). Aboriginal peoples highlight tables, 2006 census.

[18] Council of Canadian Academies (2014). Aboriginal food security in Northern Canada: an assessment of the state of knowledge.

[19] Lawn J, Harvey D (2004). Nutrition and food security in Kangiqsujuaq, Nunavik.

[20] White DM, Gerlach SC, Loring P, Tidwell AC, Chambers MC (2007). Food and water aecurity in a changing Arctic climate. Environ Res Lett.

[21] Ford JD, McDowell G, Shirley J, Pitre M, Siewierski R, Gough W (2013). The dynamic multiscale nature of climate change vulnerability: an Inuit harvesting example. Ann Assoc Am Geogr.

[22] Priest H, Usher PJ (2004). The Nunavut Wildlife Harvest Study.

[23] Kuhnlein HV, Receveur O, Soueida R, Berti PR (2008). Unique patterns of dietary adequacy in three cultures of Canadian Arctic indigenous peoples. Public Health Nutr.

[24] Kuhnlein HV, Soueida R, Receveur O (1996). Dietary nutrient profiles of Canadian Baffin Island Inuit differ by food source, season, and age. J Am Diet Assoc.

[25] Statistics Canada (2011). Census subdivision of Iqaluit, CY- Nunavut.

[26] Searles E (2010). Placing identity: town, land, and authenticity in Nunavut, Canada. Acta Boreal.

[27] Harper S, Edge VL, Ford J, Thomas MK, Pearl DL, Shirley J (2015). Acute gastrointestinal illness in two Inuit communities: Burden of Illness in Rigolet and Iqaluit, Canada. Epidemiol Infect.

[28] Bickel G, Nord M, Price C, Hamilton W, Cook J (2000). Guide to measuring household food security.

[29] Nord M, Coleman-Jensen A, Andrews M, Carlson S (2010). Household food security in the United States, 2009.

[30] Jones AD, Ngure FM, Pelto G, Young SL (2013). What are we assessing when we measure food security? A compendium and review of current metrics. Adv Nutr.

[31] Statistics Canada (2009). Household food insecurity in Canada in 2007–2008: key statistics and graphics.

[32] Tarasuk VS, Beaton GH (1999). Women’s dietary intakes in the context of household food insecurity. J Nutr.

[33] Coleman-Jensen A, Nord M, Singh A (2013). Household food security in the United States in 2012.

[34] Statistics Canada (2013). Percentage of households with food insecurity, by Province/Territory, CCHS 2011–2012.

[35] Loopstra R, Tarasuk V (2013). Severity of household food insecurity is sensitive to change in household income and employment status among low-income families. J Nutr.

[36] Sharma S (2010). Assessing diet and lifestyle in the Canadian Arctic Inuit and Inuvialuit to inform a nutrition and physical activity intervention programme. J Hum Nutr Diet.

[37] Searles EN (2010). Inuit identity in the Canadian Arctic. Ethnology/Pittsburgh.

[38] Statham S, Ford J, Berrang-Ford L, Lardeau M-P, Gough W, Siewierski R (2014). Anomalous climatic conditions during winter 2010–2011 and vulnerability of the traditional Inuit food system in Iqaluit, Nunavut. Polar Rec.

[39] Nunavut Food Security Coalition (2014). Nunavut food security strategy and action plan 2014–16.

[40] Government of Nunavut (2014). Sivumut Abluqta: stepping forward together.

